# Selection Against Maternal microRNA Target Sites in Maternal Transcripts

**DOI:** 10.1534/g3.115.019497

**Published:** 2015-08-24

**Authors:** Antonio Marco

**Affiliations:** School of Biological Sciences, University of Essex, Colchester CO4 3SQ, United Kingdom

**Keywords:** *Drosophila*, miRNA, purifying selection, polymorphisms

## Abstract

In animals, before the zygotic genome is expressed, the egg already contains gene products deposited by the mother. These maternal products are crucial during the initial steps of development. In *Drosophila melanogaster*, a large number of maternal products are found in the oocyte, some of which are indispensable. Many of these products are RNA molecules, such as gene transcripts and ribosomal RNAs. Recently, microRNAs (small RNA gene regulators) have been detected early during development and are important in these initial steps. The presence of some microRNAs in unfertilized eggs has been reported, but whether they have a functional impact in the egg or early embryo has not being explored. I have extracted and sequenced small RNAs from *Drosophila* unfertilized eggs. The unfertilized egg is rich in small RNAs and contains multiple microRNA products. Maternal microRNAs often are encoded within the intron of maternal genes, suggesting that many maternal microRNAs are the product of transcriptional hitchhiking. Comparative genomics analyses suggest that maternal transcripts tend to avoid target sites for maternal microRNAs. I also developed a microRNA target mutation model to study the functional impact of polymorphisms at microRNA target sites. The analysis of *Drosophila* populations suggests that there is selection against maternal microRNA target sites in maternal transcripts. A potential role of the maternal microRNA mir-9c in maternal-to-zygotic transition is also discussed. In conclusion, maternal microRNAs in *Drosophila* have a functional impact in maternal protein−coding transcripts.

In animals, the initial steps of embryonic development are driven by the gene products deposited by the mother into the egg. For instance, in *Drosophila melanogaster*, the anteroposterior axis is determined by the presence of maternal transcripts from genes such as *bicoid* and *nanos* (Lawrence 1992). Recently, the role of microRNAs during development has become a major area of research. MicroRNAs are small RNA molecules that regulate gene expression by targeting gene transcripts by sequence complementarity. MicroRNAs are expressed during early development (Aravin *et al.* 2003; Aboobaker *et al.* 2005), and they target other embryonic expressed gene transcripts (Enright *et al.* 2003; Lai *et al.* 2003). As a matter of fact, a number of homeotic genes detected by genetic analysis were later shown to be microRNA encoding genes [reviewed in (Marco 2012)]. Traditionally, maternal genes have been identified by genetic analysis (Lawrence 1992). However, the characterization of maternal microRNAs is particularly difficult because they are too short for standard genetic analyses. Thanks to the development of high-throughput technologies such as RNAseq and microarrays, it is now possible to isolate small RNAs directly from egg extracts. For instance, the microRNA content of mouse (Tang *et al.* 2007) and cow (Tesfaye *et al.* 2009) oocytes have been characterized with this high-throughput approach. In other cases, such as in zebrafish (Chen *et al.* 2005) and *Xenopus* (Watanabe *et al.* 2005), microRNAs appear to have a minor presence in oocytes.

Several lines of evidence suggested that, in *Drosophila*, maternally transmitted microRNAs are important. First, some microRNAs are highly abundant during early development (Ruby *et al.* 2007). Also, the enzymes responsible for microRNA biogenesis are present in the ovaries (Robinson *et al.* 2013) and microRNAs may have a role in oocyte maturation (Nakahara *et al.* 2005). Indeed, mature microRNAs have been identified in *Drosophila* unfertilized eggs (Lee *et al.* 2004, 2014; Votruba 2009). Recently, it has been shown that maternally transmitted microRNAs are adenylated during the maternal-to-zygotic transition (MZT) (Lee *et al.* 2014) .Whether maternal microRNAs have a functional impact in *Drosophila* eggs is still unknown. To identify which microRNAs are maternally transmitted, I extracted and sequenced small RNAs from *Drosophila* unfertilized eggs. To explore their potential function, I predicted their targets in maternal and zygotic gene products. The evolutionary impact of maternal microRNAs was estimated by the use of comparative genomics and population genetics.

## Materials and Methods

### Flies and egg collection

Fly stocks used in this study, with Bloomington reference number in square brackets, were: w^1118^ [#3605] and Oregon-R-modENCODE [#25221]. All flies were kept at 25° on cornmeal based media, with 12-hr light/dark cycles. Virgin females were sorted at the pupae stage to avoid any unwanted fertilization. (Previous attempts selecting for <6 hr females produced a small yet significant number of fertilized eggs.) In a population cage, I let 80−100 females to lay eggs in apple juice agar plates for 8 hr, collecting them 1 hr after dawn. Eggs were collected with a sieve and washed with saline solution. Eggs from virgin females do not degenerate even several hours after laying (Tsien and Wattiaux 1971).

### RNA extraction, sequencing, and profiling

Total RNA was extracted from eggs or early embryos with TRIzol reagent (Life Technologies), following instructions given by the manufacturer. The RNA was resuspended in nuclease-free water. For RNA sequencing, a cDNA library was generated with TruSeq Small RNA Sample Preparation Kit (Illumina). Amplified cDNA constructs were size selected in a 6% polyacrylamide gel for 145−160 bp (fragments including RNA-derived sequences of size ∼20−30 bp plus adapters). Size-selected cDNAs were purified and precipitated with ethanol, and DNA integrity was checked with TapeStation (Agilent). Samples were sequenced with Illumina MiSeq in the Genomics Core Facility at the University of Manchester. A total of 4,507,291 reads were sequenced, most of them (95.5%) deriving from ribosomal RNAs, which is expected in *Drosophila*, where the majority of small RNAs are 2S rRNA (Seitz *et al.* 2008). A total of 13,114 reads was identified as microRNA products. Sequence reads are available from Gene Expression Omnibus at the National Center for Biotechnology Information under accession no. GSE63488).

**Figure 1:**
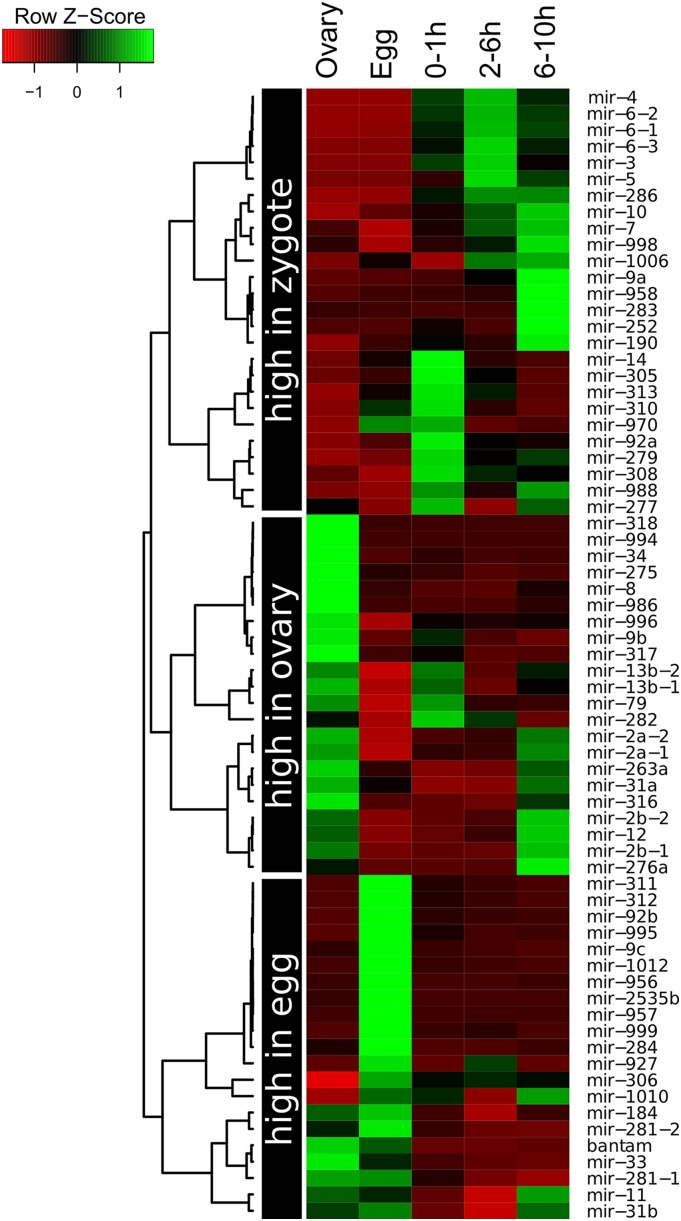
Expression profile of maternal microRNAs in *Drosophila melanogaster*. The hierarchical tree is split into three categories of microRNAs: high abundance in ovaries compared with the other stages; those that are mainly present in the unfertilized eggs; and those that have a greater expression level later during development.

Illumina MiSeq produces 50-bp sequence reads. Hence, I removed adapters with Cutadapt (https://cutadapt.readthedocs.org) and mapped the processed reads of size 18−26 bp to known microRNAs from miRBase v.20 (Kozomara and Griffiths-Jones 2014), using Bowtie v.0.12.7 (Langmead *et al.* 2009), allowing no mismatches, and considering reads mapping to up to five positions. Other RNA collections from embryos and ovaries also were analyzed: 0- to 1-hr embryos, 2- to 6-hr embryos, 6- to 10-hr embryos (Ruby *et al.* 2006), and ovaries (Czech *et al.* 2008). Expression profiling in Figure 1 was done with R (R Development Core Team 2004) by scaling the Z-scores of the heatmap across rows and generating a hierarchical tree of microRNAs with complete linkage clustering.

**Figure 2:**
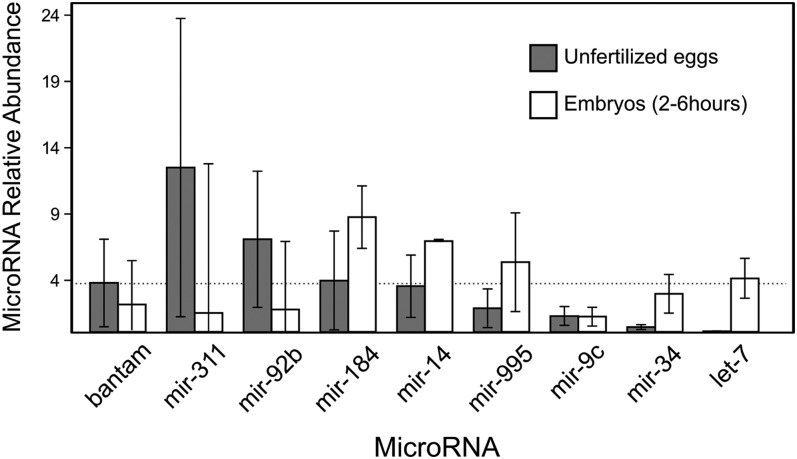
Quantification of selected maternal microRNAs in eggs and embryos. Levels of microRNA mature products in unfertilized eggs with respect to average bantam-3p levels from quantitative polymerase chain reaction assays (gray boxes; see *Materials and Methods*), and levels of microRNA mature products detected in 2- to 6-hr embryos with respect to levels in unfertilized eggs. Error bars are for three biological replicates. Dashed line indicates the levels of bantam-3p as a reference.

The presence in eggs of mature microRNAs was validated with Mir-X first-strand synthesis and SYBR quantitative reverse transcription polymerase chain reaction (qRT-PCR) assays manufactured by Clontech Laboratories, Inc. MicroRNA cDNA libraries were constructed for unfertilized eggs and 2- to 6–hr-old embryos from Oregon-R flies, following the indications from the manufacturer. Primers for microRNA-specific amplification during quantitative polymerase chain reaction (qPCR) were: let-7-5p (5′-TGAGGTAGTAGGTTGTATAGT-3′), miR-34-5p (5′-TGGCAGTGTGGTTAGCTGGTTGTG-3′), miR-311-3p (5′-TATTGCACATTCACCGGCCTGA-3′), mir-92b-3p (5′-AATTGCACTAGTCCCGGCCTGC-3′), miR-184-3p (5′-TGGACGGAGAACTGATAAGGGC-3′), miR-9c-5p (5′-TCTTTGGTATTCTAGCTGTAGA-3′), bantam-3p (5′-TGAGATCATTTTGAAAGCTGATT-3′), miR-995-3p (5′-TAGCACCACATGATTCGGCTT-3′), and miR-14-3p (5′-TCAGTCTTTTTCTCTCTCCTAT-3′). Fluorescent quantification was done in a LightCycler 96 Real-Time PCR System (Roche) for 50 cycles, cycle thresholds (Cts) were estimated with the software provided by the manufacturer with default parameters, and differences in cycle thresholds (ΔCts) calculated with U6 spliceosomal rRNA as a normalization standard. Relative expression values in Figure 2 for unfertilized eggs were calculated with respect to the average level of bantam-3p. That is: [miR]/[bantam] = 2^–ΔCt(miR)^/2^–ΔCt(bantam)^. For 2- to 6-hr embryos, the relative levels are calculated with respect to the levels in egg samples. Each amplification was performed in three biological replicates (independent egg/embryo collections) with two technical replicates each.

### MicroRNA target analysis and polymorphisms

Target analysis was based on the presence of canonical seeds in the transcripts (Bartel 2009). Canonical seed predictions have the advantage that only primary sequence information is used, so populations models (see *Results*) can be easily fitted. Maternally deposited gene transcripts are listed in the Berkeley Drosophila Genome Project Web site at http://insitu.fruitfly.org (Tomancak *et al.* 2007). Which transcripts are destabilized during MZT were identified from micro array experiments (Gene Expression Omnibus accession no. GSE13287) of Tadros *et al.* (2007) and detected probes with a >1.5-fold change in their expression level between 4- and 6-hr embryos and oocytes (Tadros *et al.* 2007). To assess whether maternal microRNAs target transcripts that are destabilized during the MTZ transition, I calculated the proportion of unstable transcripts targeted by each microRNA and compared it with the expected proportion (0.146) with a cumulative binomial test. False discovery rate was accounted by calculating q-values associated to the p-values (Benjamini and Hochberg 1995; Storey 2002).

**Figure 3:**
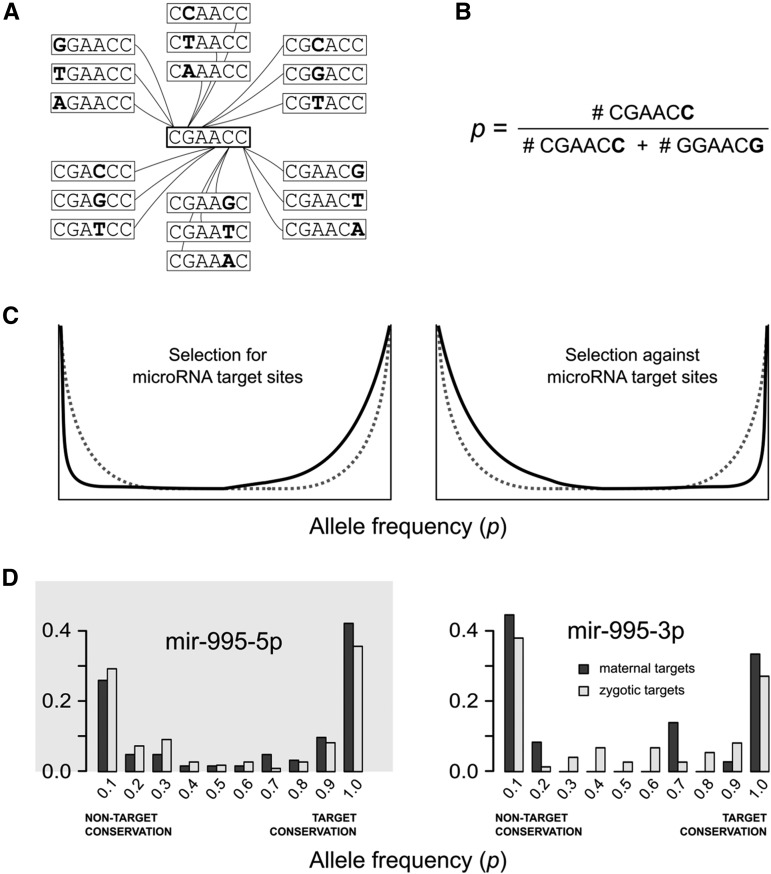
Polymorphic target sites in *Drosophila* populations. (A) Each microRNA sixmer target site has 18 one-nucleotide mutant neighbors that are themselves not target sites. (B) The allele frequency for each pair of target/nontarget sites is calculated as the proportion of target-site alleles with respect to the total number of alleles in the pair. (C) Illustration of the expected allele frequency distributions. Allele frequency distribution in a finite population is U-shaped for pairs of alleles neutral to each other (gray dashed line in both panels). If there is selection favoring target sites, distributions are expected to be shifted to the right (left panel). Conversely, if there is selection against target sites, distributions will be shifted to the left (right panel). (D) Allele frequency distribution for target sites for maternal microRNAs in maternal (dark gray) and zygotic (light gray) transcripts. Left and right panels show the distributions for 5′ and 3′ arms of mir-995, respectively. The gray shading in the left panel indicates that mir-995-5p is virtually absent in the unfertilized egg.

For the population analyses, I first mapped the single-nucleotide polymorphisms (SNPs) from the *Drosophila* Genetic Reference Panel (Mackay *et al.* 2012; Huang *et al.* 2014), available at http://dgrp2.gnets.ncsu.edu/, against the three prime untranslated region (3′ UTR) of *Drosophila melanogaster* release 5.13 (http://flybase.org). For each microRNA I defined a target sequence (sixmer) and its 18 nontarget neighbors, that is, the 18 one-nucleotide variations of the target site (Figure 3A). Every SNP that connects a target with a nontarget sixmer was further considered. 3′ UTRs with introns were discarded. For each polymorphic target site, the allele frequency distribution was calculated as the proportion of the target allele with respect to the total number of sampled individuals (isogenic lines). For each pair of alleles, both target and nontarget sites were searched in the reference genome. This way, we also account for nontarget alleles in the reference genome sequence that may be *bona fide* microRNA target sites. To study the derived allele frequencies (DAFs), I first mapped polymorphic target sites from *D. melanogaster* genome release 5.13 onto release 6 using the coordinate converter in FlyBase and then found the conserved sites in *Drosophila sechellia* by parsing the genome sequence alignment files available at UCSC Genome Browser (ftp://hgdownload.cse.ucsc.edu/goldenPath/dm6/multiz27way; Siepel *et al.* 2005) by using custom-made PERL scripts. Maternal microRNAs considered in the DAF analysis were mature sequences highly abundant in unfertilized eggs: bantam-3p, mir-1010-3p, mir-10-5p, mir-11-3p, mir-14-3p, mir-184-3p, mir-263a-5p, mir-276a-3p, mir-279-3p, mir-281-2-5p, mir-305-3p, mir-305-5p, mir-306-5p, mi, -313-5p, mir-318-3p, mir-31a-5p, mir-33-5p, mir-8-3p, mir-956-3p, mir-995-3p, mir-999-3p, and mir-9c-5p. Nonmaternal microRNAs were those not expressed in any tissue/stage according to the information available from miRBase. These microRNAs (with available SNP information) were: mir-3644-5p, mir-4941-3p, mir-4944-3p, mir-4944-5p, mir-4963-5p, mir-4967-5p, mir-4972-3p, mir-4979-5p, mir-4982-3p, and mir-4985-3p.

### Data availability

Table S1 lists all microRNAs detected in unfertilized eggs and their read counts. File S1 contains the frequency of derived alleles used to generate Figure 6. Raw sequencing data is available from Gene Expression Omnibus (GEO) with accession number GSE63488.

## Results

### Mature microRNAs are maternally deposited in the egg

To identify maternal microRNAs in *Drosophila*, I first characterized RNAs from unfertilized eggs with high-throughput sequencing (see *Materials and Methods*). The most abundant microRNAs in unfertilized eggs were produced by *mir-92b*, *mir-184*, the *mir-310/mir-311/mir-312/mir-313* cluster, and *bantam* genes, which accounted for over half of the microRNA reads. Table 1 shows microRNA loci producing more than 13 reads (1‰ of the microRNA-associated reads). A full list of detected microRNAs with their read counts is available in Supporting Information, Table S1. The dataset was screened for new microRNAs as described previously (Marco *et al.* 2010, 2013a; Marco and Griffiths-Jones 2012), but no new microRNAs were found. This tell us that maternal microRNAs are already known in *Drosophila*.

**Table 1:** Maternal microRNAs in *Drosophila melanogaster*

MicroRNA Transcript*^a^*	Reads per miRNA	Total Reads (%)*^b^*
mir-310/311/312/313	356/2012/1661/82	4111 (31.3)
mir-92a/92b	172/2109	2281 (17.4)
mir-184	1377	1377 (10.5)
mir-9c/306/79/9b	1064/154/4/132	1354 (10.3)
bantam	1204	1204 (9.2)
mir-995	624	624 (4.8)
mir-14	411	411 (3.1)
mir-275/305	90/269	359 (2.7)
mir-998/11	5/205	210 (1.6)
mir-8	209	209 (1.6)
mir-2b-2/2a-1/2a-2	85/25/19	129 (1.0)
mir-279/996	61/56	117 (0.9)
mir-2b-1	92	92 (0.7)
mir-281-2/281-1	66/13	79 (0.6)
mir-4969/999	0/69	69 (0.5)
mir-33	62	62 (0.5)
mir-263a	59	59 (0.4)
mir-10	28	28 (0.2)
mir-2c/13a/13b-1	0/0/27	27 (0.2)
mir-13b-2	26	26 (0.2)
mir-970	26	26 (0.2)
mir-1012	25	25 (0.2)
mir-31a	23	23 (0.2)
mir-9a	21	21 (0.2)
mir-309/3/286/4/5/6-1/6-2/6-3	0/1/15/1/1/1/1/1	21 (0.1)
mir-956	20	20 (0.2)
mir-276a	18	18 (0.1)
mir-994/318	2/14	16 (0.1)
mir-1010	14	14 (0.1)

a MicroRNAs clustered in the genome (<10 kb).

b Percentage over total number of reads mapping to microRNAs.

In a recent report, Narry Kim and collaborators identified maternally transmitted microRNAs in *Drosophila* and demonstrated that they are targeted to degradation during MZT by adenylation via Wispy (Lee *et al.* 2014). Their set of maternal microRNAs is virtually identical to the set here described. Overall, the read counts from both datasets are highly correlated (R^2^ = 0.62; p < 0.001, Figure S1A). The overlap for the top N-th most abundant microRNAs between both datasets is highly significant (Figure S1B). Specifically, the microRNAs here described as maternal in Table 1 (more than 13 reads) are the top 35 mature sequences, overlapping with 26 microRNAs from the top 35 microRNAs of Lee *et al.* (2014) (74.3%; p = 0.00031, Figure S1B). Additionally, the read counts form this study and a recent report by Ninova *et al.* (2015), which uses the same protocol for RNA extraction and sequencing, are highly correlated (R^2^ = 0.86; p < 0.001; Figure S1C). All these observations support the high confidence of the maternal microRNA set here described.

Figure 1 compares the relative expression of maternal microRNAs in the ovary, unfertilized eggs and early stages of development. From this comparison, three types of maternal microRNAs can be distinguished. First, some maternal microRNAs are highly expressed in the ovary. A second class consists on microRNAs that are found primarily in the unfertilized egg. Third, a large proportion of maternal microRNAs is also transcribed later on during development. These groups are referred as “high in ovary,” “high in egg,” and “high in zygote” maternal microRNAs in Figure 1. Some of these microRNAs were detected at very low levels, and whether they are *bona fide* maternal microRNAs may need further evidence.

To further confirm the presence of maternal microRNAs in unfertilized eggs, I validated the presence of highly abundant mature products by qPCR (see *Materials and Methods*). Figure 2 shows the relative abundance of selected microRNAs (with respect to the average level of bantam-3p). Although the microRNA level varies substantially across biological replicates, the presence of seven of the maternal microRNAs here described is validated (bantam-3p, mir-311-3p, mir-92b-3p, mir-184-3p, mir-14-3p, mir-995-3p, and mir-9c-5p), although the levels of the latter two were relatively low. Furthermore, the level of mir-34-5p, which has been reported to be maternally transmitted (Soni *et al.* 2013), was very low, in agreement with this and other investigations (see *Discussion*). The conserved microRNA let-7-5p was used as a negative control, as it was not detected in unfertilized eggs. In the qPCR analysis, let-7-5p was not amplified in unfertilized eggs (Figure 2). I further measured the relative levels of maternal microRNAs in later stage embryos (2−6 hr). In concordance to the high-throughput sequencing analysis presented in Figure 1, bantam-3p, mir-311-3p, and mir-92-3p were more abundant in the unfertilized egg than in the developing embryo. In contrast, mir-14-3p was greater expressed in the embryo than in the egg. However, for mir-184-3p and mir-995-3p, the pattern was not consistent between RNAseq and qPCR. The differences were not significant. Both mir-34-5p and let-7-5p were highly abundant in developing embryos, further supporting that they are virtually absent from the unfertilized egg and expressed from the zygotic genome at later stages during development.

### Intronic maternal microRNAs hosted in maternal protein−coding genes

In a previous work, I observed that female-biased microRNAs tend to be produced from introns of female-biased protein coding transcripts (Marco 2014). For instance, mir-92a is highly expressed in females, and it is encoded within the *jigr1* gene, which is maternally deposited in the egg. Here I show that mir-92a is also maternal. To further explore the relationship between maternal microRNAs and the maternal deposition of overlapping genes, I compared the expression pattern of intronic maternal microRNAs and the host protein coding gene. Table 2 lists 12 maternal microRNA clusters hosted in protein coding genes. For nine of these host genes, there are *in situ* hybridization experiments (Tomancak *et al.* 2002, 2007), and eight of them are maternally loaded. Because 55.8% of genes in this dataset are shown to produce maternally deposited transcripts, our set of host genes is statistically enriched for maternal products (p ∼ 0.044; binomial test). There is no information from high-throughput *in situ* hybridization analyses for *grp*, but it is known to be present in unfertilized oocytes (Fogarty *et al.* 1997). The other two host genes have no expression information available at FlyBase. From this analysis I conclude that intronic maternal microRNAs are frequently produced from introns of maternally deposited gene transcripts.

**Table 2:** Maternal microRNA loci within protein-coding genes

MicroRNA Cluster	Host Gene	Protein-Coding Gene Maternal?
mir-995	cdc2c	Yes
mir-11/998	E2f	Yes
mir-92a	jigr1	Yes
mir-999	CASK	Yes
mir-281-1/281-2	Oda	Yes
mir-970	Tomosyn	Yes
mir-2b-2/2a-1/2a-2	spi	Yes
mir-13b-2	CG7033	Yes
mir-9c/306/79/9b	grp	Yes*^a^*
mir-33	HLH106	No expression information
mir-1012	Lerp	No expression information
mir-1010	SKIP	No

a Detected in the oocyte.

### Maternal microRNAs in the MZT transition

As shown in Figure 1, a significant fraction of maternal microRNAs have a lower expression when zygotic transcription starts. One possibility is that some of these maternal microRNAs have a role in destabilizing maternal transcripts during the MZT. A similar role has been described for early expressed zygotic microRNAs in *Drosophila* (Bushati *et al.* 2008) and other species such as zebrafish (Giraldez *et al.* 2006). I predicted target sites for each maternal microRNAs in stable and unstable maternal transcripts during MZT (Tadros *et al.* 2007). Table 3 shows maternal microRNAs targeting more unstable maternal transcripts than expected by chance (false discovery rate < 10%). Two of the microRNAs, mir-283 and mir-277, were detected at very low levels in unfertilized eggs (Table S1) and have a greater expression level later on during embryonic development (Figure 1). It is possible that these microRNAs contribute to the destabilization of maternal transcripts, but probably as zygotic microRNAs. Other sets of microRNAs that may contribute to transcript clearance during MZT are the mir-310 and mir-92 families. They both share the same seed sequence (which determines the targeted transcripts). These are also zygotic microRNAs expressed very early during development. Last, the microRNA-9 family also targets unstable maternal transcripts. Members of the mir-9 family, particularly mir-9c, are particularly abundant in unfertilized eggs but lower expressed in early embryos (Figure 1). This indicates that mir-9 may be the first case of a maternal microRNA contributing to the degradation of maternal transcripts during MZT. In summary, some maternally deposited microRNAs have a potential role in destabilizing maternal transcripts.

**Table 3:** Maternal microRNAs targeting unstable transcripts during maternal-to-zygotic transition

MicroRNA	Unstable Targets	Stable Targets	Proportion Unstable Transcripts*^a^*	q-Value*^b^*
mir-283-5p	179	805	0.182	0.018
mir-277-3p	116	497	0.189	0.026
mir-9a/b/c-5p	50	166	0.232	0.036
mir-92a/b-3p; mir-310/311/312/313-3p	74	313	0.191	0.096

a Expected proportion is 0.146.

b Binomial test.

### Maternal protein−coding transcripts are selected against target sites for maternal microRNAs

If maternal microRNAs have a functional impact on maternal transcripts, these transcripts should have a different target repertoire compared with zygotic transcripts. I estimated how many maternal and zygotic transcripts are targeted by maternal microRNAs. Overall, 73% of maternal transcripts and 63% of zygotic transcripts have canonical seed target sites for maternal microRNAs. However, for transcripts from genes with a recent evolutionary origin, that is, that they originated in the *Drosophila melanogaster* lineage, maternal transcripts were less likely to be targeted by maternal microRNAs than zygotic transcripts: 50.7 ± 0.5% of maternal transcripts have canonical target sites for maternal microRNAs, whereas this percentage is 52.6 ± 0.4% for zygotic transcripts (p ∼ 0.004; *t*-test). Although the difference is small, the observation that evolutionarily young maternal genes have a relatively lower proportion of targets for maternal microRNAs than zygotic genes suggests purifying selection against microRNA targets. In other words, if there was no selection against microRNA targets, we would expect a similar proportion of target sites between maternal and zygotic transcripts.

To test whether there is selection against maternal microRNA target sites, we should evaluate population data. To do so, I first constructed a model of microRNA target mutation as follows (see Figure 3A): 1) a target site is defined as any six-nucleotide sequence (sixmer) in a 3′ UTR complementary to the seed region (Bartel 2009) of a microRNA; 2) any target site has 18 mutant neighbors, which are one nucleotide mutation apart from the canonical target, and are not themselves targets; 3) only polymorphic sites in which one of the alleles is a target site and the other a nontarget are further considered in this analysis. Allele frequency is here defined as the proportion of the target allele (*p* in Figure 3B). For instance, an allele frequency of 0.8 means that 80% of the sampled individuals have the target site at a given position and 20% have a nontarget mutant neighbor. Conversely, an allele frequency of 0.3 will indicate that the nontarget neighbor is more frequent (70%) than the target allele (30%). Population genetics theory (Crow and Kimura 1970; Nei 1975) predicts that, in a finite population, two alleles neutral to each other will have a symmetric U-shaped distribution, that is, most individuals will be homozygous for one of the alleles. However, if there is a selective pressure to conserve a target site, the distribution will be shifted to the right. On the other hand, if selection is against a target site allele, the distribution will be shifted to the left (see Figure 3C). A symmetric U-shape distribution is not expected if other evolutionary forces are in place (for instance, mutation bias, or background selection produced by purifying selection on neighboring sites). Hence, to estimate the selective pressure for, or against, a microRNA target site in maternal transcripts, we need an empirical expected distribution of allele frequencies. Therefore, I calculated the allele frequency at target sites in zygotic transcripts, in which maternal microRNAs have no (or little) influence. By comparing the allele frequency distribution of target sites between maternal and zygotic transcripts, we can estimate the relative selective pressure on microRNA target sites in maternal with respect to zygotic transcripts.

**Figure 4:**
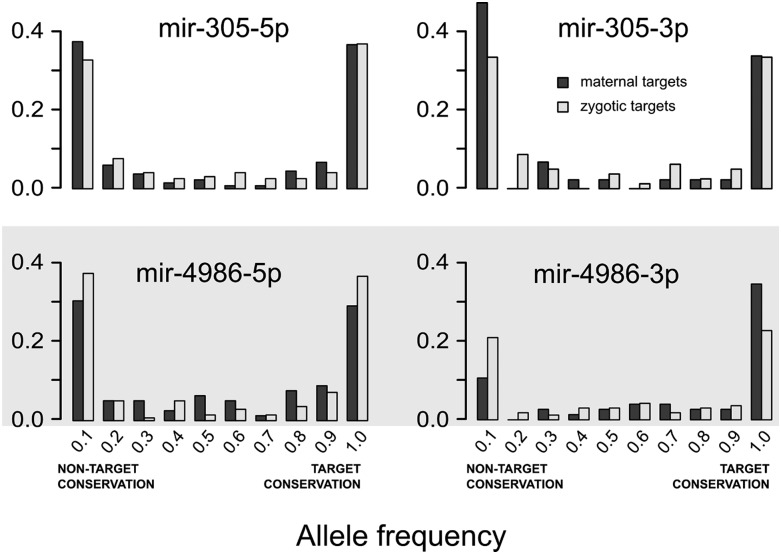
Allele frequency distribution for target sites of maternal microRNAs. Distribution of targets for maternal and zygotic transcripts for mir-305 (both arms are highly present in the egg) and mir-4986 (gray box, neither of the arms was detected in the egg).

Figure 3D shows the case for *mir-995* microRNA products. One of them, mir-995-3p, is abundant in unfertilized eggs whereas the alternate arm, mir-995-5p, is virtually absent in eggs. The allele frequency distribution in maternal transcripts is shifted to the left with respect to zygotic transcripts in mir-995-3p. That is not the case for mir-995-5p. In other words, there is a preference for alleles that are nontargets of maternal mir-995-3p, but not for the nonmaternal mir-995-5p. Both arms of mir-305 are present at high levels in unfertilized eggs. Figure 4 (top) shows the allele frequency distribution for their targets, and both arms show evidence of selective pressure against maternal microRNA target sites. As a counterexample, Figure 4 (bottom) shows the allele frequency distribution of a microRNA for which none of the arms was detected in unfertilized eggs: mir-4986. Consistently, none of the microRNA products showed evidence of selection against target sites.

**Figure 5:**
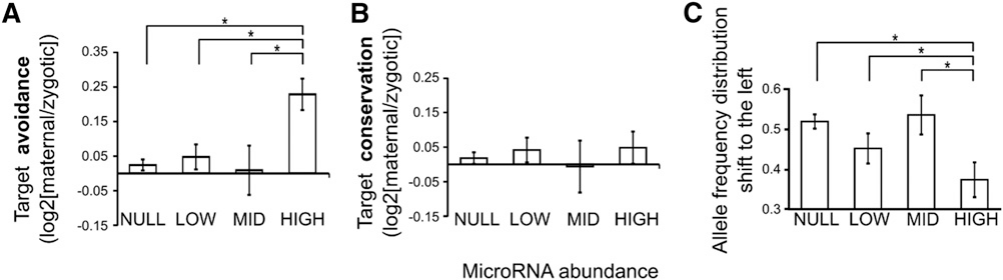
Maternal microRNA target avoidance. (A) Target avoidance (see *Results*) for microRNAs with differences abundances in the unfertilized egg (NULL, not detected; LOW, less than 0.1% of the set; MID, between 0.1 and 1%, HIGH, more than 1%). Error bars represent the SEM. Asterisks show statistically significant differences (p < 0.01) for *t*-test with unequal variances. (B) Target conservation for microRNAs with differences abundances in the unfertilized egg. (C) Distribution shifting to the left of allele frequency distribution in maternal transcripts with respect to zygotic transcripts.

To explore whether this pattern is a general feature of maternal microRNAs I defined “target avoidance” as the log2 ratio of the number of target sites with an allele frequency smaller than 0.1 (that is, the frequency of sites where >90% of alleles are the nontarget sequence) between maternal and zygotic transcripts. In this context, positive values indicate that targets for a specific microRNA tend to be “avoided” by maternal transcripts, that is, there is selection against target sites for maternal microRNAs in maternal transcripts. Figure 5A is a bar plot of target avoidance values for different levels of microRNA abundance in the egg. Maternally deposited coding transcripts tend to avoid some target sites for highly abundant maternal microRNAs (with respect to zygotic transcripts). Differences were statistically significant (Figure 5A). In a similar manner, I defined “target conservation” as the log2 ratio of the number of target sites with allele frequency greater than 0.9 between maternal and zygotic transcripts. A positive value indicates that target-sites are preferentially conserved in maternal transcripts. Figure 5B shows these values for different microRNA abundances. Overall, maternal transcripts conserve some target sites, but there is not a distinctive profile between maternal and nonmaternal microRNAs (Figure 5B).

We also can compare the whole-allele frequency distribution and evaluate whether the distribution for maternal transcripts is shifted to the left with respect to that of zygotic transcripts (as suggested in Figure 3C, right panel). To do so I performed a one-tail Kolmogorov-Smirnov test for each pair of allele frequency distributions and evaluated whether the distribution of maternal alleles was shifted to the left compared with the zygotic allele distribution, that is, whether there is a preference for the nontarget allele in maternal transcripts. The lower the p-value, the larger the shift to the left. This measure is not independent from that in Figure 5A, and I use it here as an alternative method to evaluate target avoidance. Figure 5C plots the Kolmogorov-Smirnov p-value and shows that these are significantly lower for microRNAs that are abundant in unfertilized eggs. The allele frequency distributions of maternal microRNA target sites in maternal transcripts are, therefore, biased toward the nontarget allele.

**Figure 6:**
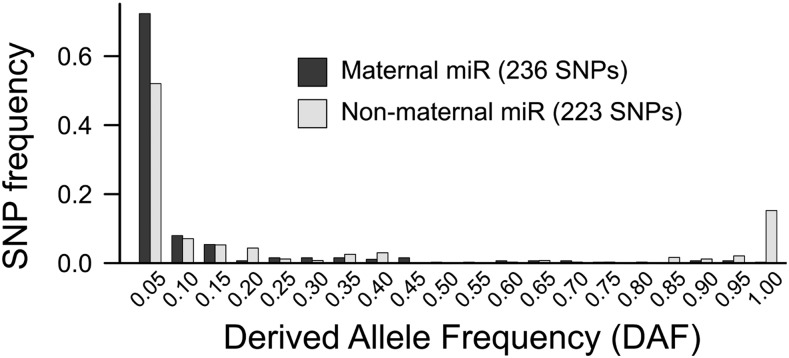
Derived allele frequency distribution of microRNA target sites. Shown is the allele frequency distribution of single-nucleotide polymorphisms that are microRNA targets whose predicted ancestral state was a nontarget site. The frequency distribution for maternal microRNA target sites is plotted in dark gray boxes, and the distribution for nonmaternal microRNA target sites in light gray boxes.

The comparison of two allele frequency distributions has been very useful to detect selection and/or mutational biases (Eyre-Walker *et al.* 2006; Galtier *et al.* 2006). Nevertheless, it is difficult to infer directionality in the evolutionary process because we do not know which one was the ancestral allele. One way to circumvent this issue is to compute the DAF distribution, which is the allele frequency distribution of alleles that were not ancestral. The comparison of DAF distributions has been of much use to infer selection in genes (Yngvadottir *et al.* 2009) and in regulatory sites (Sethupathy *et al.* 2008). I computed the DAF distribution for nontarget sites to explore signatures of selection against target sites in maternal transcripts. First, I cataloged, among all target/nontarget pair of alleles analyzed in this study, those sites that were conserved in *D. sechellia*, which diverged from the *D. melanogaster* lineage about 2 million years ago. Then, I selected those polymorphic sites that are nontarget sites in *D. sechellia* and, assuming that that was the ancestral state, I plotted the frequency distribution of the target alleles in *D. melanogaster*. As maternal microRNAs, I selected highly abundant mature sequences. As nonmaternal, I selected microRNAs that were not present in the egg but also not expressed in other tissues (see *Materials and Methods*) . Figure 6 compares the DAF for ancestral nontarget sites in maternal transcripts between maternal and nonmaternal microRNAs. In agreement with the previous analyses, the derived allele frequencies are smaller for maternal than for nonmaternal microRNA targets. The difference between the two distributions was significant (p < 0.0001; Kolmogorov-Smirnov test). Also, there was an excess of singletons in maternal (67 of 236) compared with nonmaternal (44 of 223) microRNA target sites (p = 0.0304; χ^2^ test). The whole dataset is available in File S1. That indicates that selection may favor the derived allele, that is, the nontarget allele. In conclusion, different analyses suggest purifying selection against maternal microRNA target sites in maternal transcripts.

## Discussion

This study characterizes microRNA products from *Drosophila* unfertilized eggs. I validated seven of these microRNAs by qPCR. The presence of microRNAs in unfertilized oocytes have been described in mice (Tang *et al.* 2007). However, it has been shown that microRNA activity is suppressed in mice oocytes, indicating that maternally deposited microRNAs may not have a defined function in this species (Ma *et al.* 2010; Suh *et al.* 2010). Here I show evidence for *Drosophila* maternal microRNA activity as they have an impact in the evolution of potential target sites in maternal microRNAs (Figure 3, Figure 4, Figure 5, and Figure 6).

One of the most abundant maternal microRNAs, mir-184, has been already described in freshly laid *Drosophila* eggs (Iovino *et al.* 2009). Also, the *mir-184* gene has an important role during oocyte development as well as in early development (Iovino *et al.* 2009). Another maternal microRNA gene, *mir-14*, seems to be involved in transcriptional silencing of transposable elements in the germline (Mugat *et al.* 2015). On the other hand, the conserved microRNA mir-34 has been also described as a maternal microRNA (Soni *et al.* 2013) but it has only one read copy in our dataset, and it has not been detected in two other independent high-throughput screens (Lee *et al.* 2014; Ninova *et al.* 2015). The level of mir-34-5p was also very low in specific qPCR assays (Figure 2). All these findings suggest that either mir-34 is a very low copy maternal microRNA, or that it is rapidly degraded after egg deposition/activation.

Another maternal microRNA gene, *mir-9c*, is necessary to regulate the number of germ cells (Kugler *et al.* 2013). Indeed, is the maternal loss of *mir-9c* what produces this phenotype (Kugler *et al.* 2013). This microRNA is hosted within a maternally deposited gene, *grapes* (Table 2). Here I show that mir-9c-5p targets more unstable transcript during the MZT than expected by chance (Table 3), which indicates that mir-9c-5p may have a role during maternal transcript clearance during the initial steps of development. A similar role has been described for zygotically transcribed microRNAs (Bushati *et al.* 2008).

Other maternally deposited microRNAs derive from the *mir-310/mir-313* cluster. This cluster is highly conserved in the *Drosophila* lineage (Marco *et al.* 2013b), although it may have originated in insects (Ninova *et al.* 2014), and is evolutionarily related with the (also maternal) *mir-92a/mir-92b* cluster (Lu *et al.* 2008; Ninova *et al.* 2014). Mature products from the orthologous *mir-310/311/312/313* and *mir-92a/92b* clusters in *Drosophila virilis* have been detected at high levels during the first 2 hr of development, suggesting that these microRNAs are also maternally deposited in this species [Table S2 in (Ninova *et al.* 2014)]. Interestingly, some maternal microRNAs have other functions later on during development. MicroRNAs from the *mir-310/311/312/313* cluster are known to be involved in male gonad development (Pancratov *et al.* 2013). Recently, Ranz and collaborators found that mir-310/mir-313 microRNAs show male biased expression pattern at the onset of metamorphosis (Yeh *et al.* 2014). On the other hand, *mir-92a* is expressed in the adult, and it is involved in leg morphology (Arif *et al.* 2013). Some other maternal microRNAs have roles unrelated with embryonic development, such as *mir-14*, which regulates insulin production (Xu *et al.* 2003); *mir-279*, involved in the circadian clock (Luo and Sehgal 2012); or *mir-8*, associated to abdominal pigmentation (Kennell *et al.* 2012), to name but a few cases. Altogether, these examples show that maternal microRNAs frequently have other functions at different developmental stages and/or tissues.

MicroRNA target avoidance has been observed in *Drosophila* (Stark *et al.* 2005), as well as in mice (Farh *et al.* 2005) and humans (Sood *et al.* 2006; Chen and Rajewsky 2006). Here I detect a similar pattern in *Drosophila* eggs, in which maternal transcripts tend to avoid target sites for maternal microRNAs. Alternatively, a lower number of target sites in maternal transcripts may be explained as an early degradation of transcripts with conserved target sites and therefore not detected in early embryos. However, in *Drosophila*, microRNA-mediated transcript degradation happens a few hours after microRNA-mediated repression (Djuranovic *et al.* 2012). Maternal transcripts are detected from 0- to 2-hr-old embryos, and they are unlikely to have had microRNA-mediated transcript degradation. The microRNA genes studied in that paper were *mir-9b*, *mir-279*, and *bantam*, all of which were detected in this study as maternal.

If microRNAs are likely to have a function in maternal transcripts, why we observe selection against target sites? I suggest the following explanation. A microRNA that is maternally deposited and targets several maternal microRNAs may have a function, for instance, induce the programmed degradation of maternal transcripts during MZT. However, there are hundreds of other maternal transcripts that should not be targeted. This situation creates a conflict in which functional interactions must be conserved, but new interactions that potentially impair existing regulatory networks should be avoided. In this context, most maternal transcripts will be selected against target sites for maternal microRNAs. It is likely that this conflict also happens in other tissues and species and probably will also affect transcription factor−mediated regulation. How much selection against regulatory sites affects genome evolution is not yet known, and more studies need to be done.

The main advantage of working with microRNAs to study evolution at the population level is that we can predict the impact of single-point mutations in both the microRNAs and their targets. This is not yet possible with other gene regulators, such as transcription factors. I introduce a simple mutation model to study target/nontarget allele pairs and propose that comparing the allele frequencies at target sites between two groups of targeted genes can be use to infer selective pressures on microRNA target sites. The use of population genetics to study the evolutionary dynamics of microRNA target sites is still an underdeveloped research area. Despite the limitations of the model here introduced, it has been proved to be useful to detect selection at microRNA target sites. I anticipate that more accurate models and the analyses of bigger sets of microRNA target sites will shed light on how microRNA function diversify and, more generally, how gene regulation evolves.

Overall, this paper describes three features of maternally transmitted microRNAs: 1) they are often produced from introns of maternally deposited transcripts; 2) they can be zygotically transcribed and have other functions during development; and 3) maternal transcripts tend to avoid target sites for maternal microRNAs. Additionally, I suggest that mir-9c may be involved in maternal transcript clearance during MZT. These observations indicate that some maternal microRNAs may have a function but are potentially damaging to the normal function of other maternal genes. Therefore, selective pressures may prevent maternal transcripts to be targeted by maternal microRNAs.

## 
